# Exploiting cooperative pathogen behaviour for enhanced antibiotic potency: A Trojan horse approach

**DOI:** 10.1099/mic.0.001454

**Published:** 2024-04-30

**Authors:** Alper Mutlu, Emily J. Vanderpool, Kendra P. Rumbaugh, Stephen P. Diggle, Ashleigh S. Griffin

**Affiliations:** 1 Department of Biology, University of Oxford, Oxford, UK; 2 Texas Tech University Health Sciences Center, Lubbock, USA; 3 Center for Microbial Dynamics and Infection, School of Biological Sciences, Georgia Institute of Technology, Atlanta, Georgia, USA

**Keywords:** antimicrobial resistance, cooperation, cheat, trojan cheat, microbial social evolution, quorum sensing, virulence

## Abstract

Antimicrobial resistance poses an escalating global threat, rendering traditional drug development approaches increasingly ineffective. Thus, novel alternatives to antibiotic-based therapies are needed. Exploiting pathogen cooperation as a strategy for combating resistant infections has been proposed but lacks experimental validation. Empirical findings demonstrate the successful invasion of cooperating populations by non-cooperating cheats, effectively reducing virulence *in vitro* and *in vivo*. The idea of harnessing cooperative behaviours for therapeutic benefit involves exploitation of the invasive capabilities of cheats to drive medically beneficial traits into infecting populations of cells. In this study, we employed *Pseudomonas aeruginosa* quorum sensing cheats to drive antibiotic sensitivity into both *in vitro* and *in vivo* resistant populations. We demonstrated the successful invasion of cheats, followed by increased antibiotic effectiveness against cheat-invaded populations, thereby establishing an experimental proof of principle for the potential application of the Trojan strategy in fighting resistant infections.

## Introduction

The fight against microbial pathogens is almost completely reliant on the development of new antibiotics. However, the long-term effectiveness of this strategy is threatened due to the evolution of resistance by pathogens [1,2]. Hence, there is a growing interest in developing ‘evolution-proof’ strategies to fight against infections [3,6]. The idea of utilizing social cheats as therapeutic agents has been proposed based on theoretical and empirical studies on cooperative virulence and cheat invasion [4,6]. However, experimental evidence supporting the effectiveness of cheat therapies is limited.

Bacterial virulence is a complex phenomenon that frequently relies on cooperative interactions facilitated by the release of public goods [7,9]. This cooperative behaviour can be targeted directly, similar to conventional drug treatments [3,6], or it can be exploited by utilizing cheats [4,6]. Cheats are cells that do not contribute to the production of public goods but still benefit from cooperation. Consequently, cheats can invade populations by capitalizing on their fitness advantage [9,12]. Furthermore, cheat invasion is expected to lead to a reduction of virulence due to reduced cooperation within the population [4].

**Fig. 1.**
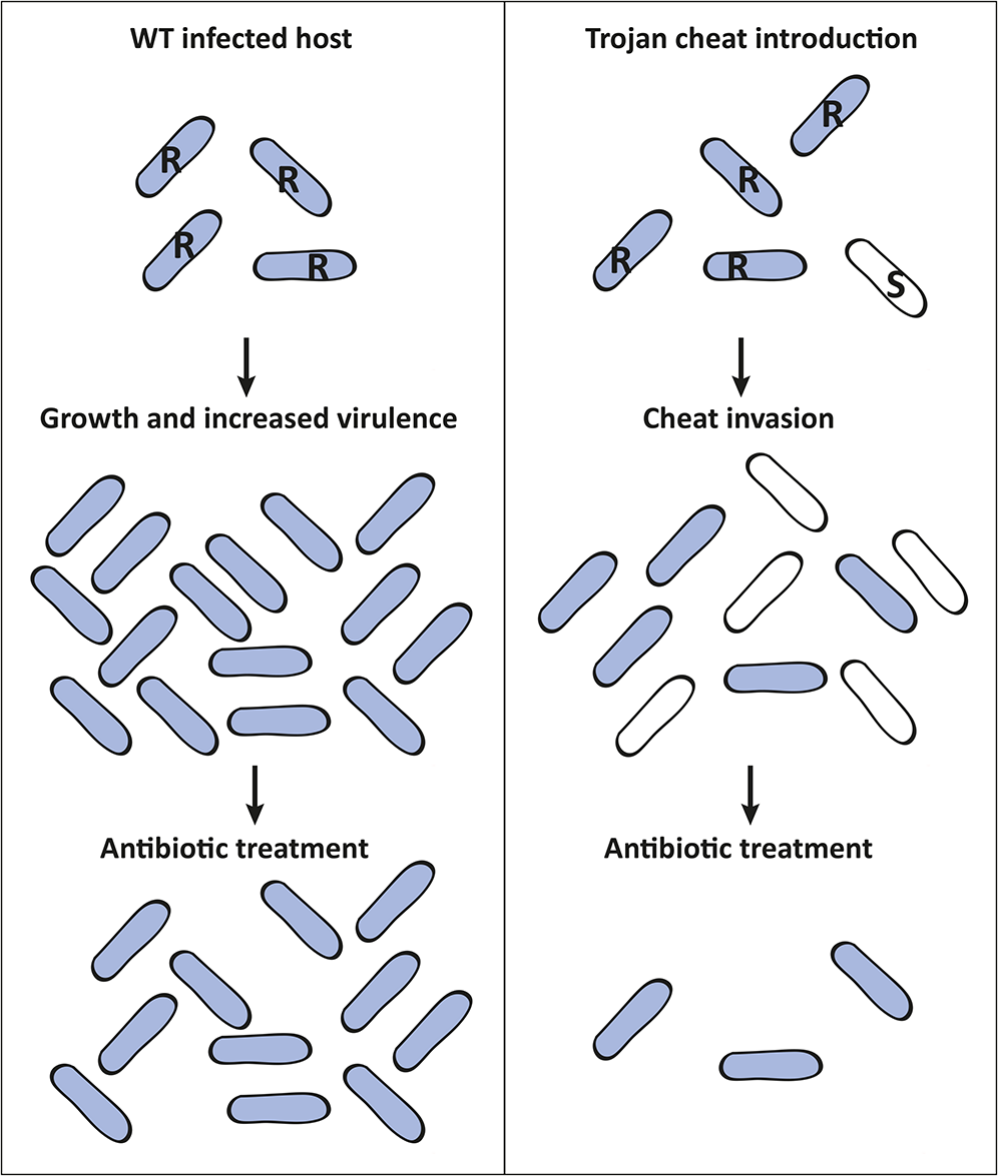
Trojan horse cheat intervention strategy to fight resistant infections. Antibiotic-resistant populations multiply and cause severe infections (left panel). A Trojan horse approach can be used to introduce medically beneficial alleles (e.g. antibiotic sensitivity) into antibiotic-resistant pathogenic populations to reduce total bacterial load and virulence (right panel). Blue cells show antibiotic-resistant (R), and white cells show antibiotic-sensitive (S) subpopulations.

Empirical evidence of cheat invasion in *Pseudomonas aeruginosa* has been demonstrated through *in vitro* experiments [11,14]. Moreover, studies by Harrison *et al.* [15] and Rumbaugh *et al.* [16] demonstrated the invasion potential of *P. aeruginosa* cheats and subsequent reduction in virulence using animal infection models. Additional evidence of cheat invasion has been shown using animal models of *Salmonella enterica* serovar *Typhimurium* and *Staphylococcus aureus* infections [17,18]. Expanding on these findings, cheats in pathogen populations can be further manipulated to become Trojan cheats: strains which act as vehicles for medically beneficial alleles such as antibiotic sensitivity or genes which quench quorum senging (QS) signals (Fig. 1). The concept of employing cheats as Trojan horses has gained traction over the past 15 years [4,5, 16] as a way of ‘socially engineering’ populations, similar to genetic engineering at the individual genome level. The primary objective of social engineering is to minimize the total bacterial burden in the targeted population. Even if resistant strains survive, the number of infectious individuals may decrease to levels susceptible to elimination by the host’s immune system. Moreover, antibiotic sensitive strains can always be re-introduced after the initial treatment [4].

Why would cells fail to evolve resistance to cheat intervention strategies in the same way that they evolve resistance to antimicrobial treatments? The answer to this question is well-understood from evolutionary theory. The simplest way to avoid exploitation is to refrain from engaging in cooperative actions [5,19]. However, by avoiding cheating, bacterial cells become less virulent, and the overall goal is still achieved. Additionally, once invaded by cheats, cooperators are unable to invade a population of cheats because rare cooperators face a fitness disadvantage on a local scale. These individuals bear all the costs to provide a valuable public good, while the benefits are enjoyed by neighbours who bear none of the costs [13]. Therefore, a cooperative (and consequently more virulent) cell must overcome significant evolutionary and mechanistic constraints to resist cheating.

Here we present a proof of principle for application of cheats as part of a Trojan horse strategy to drive a selected beneficial allele to populations of cooperators. We used cheats of *P. aeruginosa,* defective in QS signalling, to invade and introduce antibiotic sensitivity in populations of resistant cooperators to improve antibiotic effectiveness. First, we demonstrated the invasive ability of a Trojan strain *in vitro* using head-to-head competition assays in both liquid and viscous media (increased viscosity and spatial structuring are expected in real-life infections; and has been shown to affect cooperator cheat dynamics) [20,21]. We then exposed the Trojan invaded populations to antibiotic selection and showed improved antibiotic efficacy. Finally, we provided evidence of a successful application of the Trojan invasion strategy to improve antibiotic efficiency using a murine chronic wound infection model.

## Methods

### Bacterial strains and media

For all experiments, wild-type *P. aeruginosa* PA14 strain was used as the parental strain. The Trojan strain was an isogenic QS signal blind mutant of PA14 generated by insertion mutagenesis in the *lasR* locus *lasR*::Gm (PA14Δ*lasR*) [16]. A multi-drug resistance plasmid, pAMBL2 [22] was used to obtain antibiotic-resistant variants (Table 1). To generate resistant PA14 and PA14Δ*lasR* strains an electroporation protocol was used [23].

**Table 1.** Strains used in this study

Strain abbreviation	Strain description
PA14	Parent Strain
Trojan/ Trojan cheat	PA14Δ*lasR*
PA14^R^	PA14, pAMBL2
Trojan^R^	PA14Δ*lasR,* pAMBL2

To grow cultures, cells were inoculated from frozen stocks into fresh King’s broth media [KB, 20 g proteose peptone No3, 10 ml glycerol, 1.5 g K_2_HPO_4_.3H_2_O and 1.5 g MgSO_4_.7H_2_O, per litre of dH_2_O]. Then cultures were incubated overnight at 37 °C with 200 r.p.m. shaking. Next, the overnight cultures were centrifugated, supernatant was discarded, and the pellet was washed twice in M9 minimal salts media [6.8 g Na_2_HPO_4_, 3 g KH_2_PO_4_, 0.5 g NaCl, and 10 g NH_4_Cl, per litre of dH_2_O] to remove residual carbon sources from the KB media. Cell density was assessed by measuring absorbance at 600 nm, and each set of cultures were standardized to the same density before the competition assays.

### *In vitro* competition assays in defined QS media

To determine the relative fitness of cheats, a defined QS medium (QSM) was used. PA14 and Trojan strains were inoculated in a 10 : 1 ratio in 50 ml falcon tubes containing 2 ml QSM [1 % BSA w/v and 0.1 % CAA w/v in M9 solution] as described previously [11,24]. Then, culture tubes were incubated at 37 °C for 24 or 48 h with shaking at 50 r.p.m. To control for social effects, both strains were also grown in monocultures. Relative frequencies at the start and at the end of experiments were determined by plating on LB agar [25 g LB Miller agar per litre of dH_2_O]. After 24 h incubation at 37 °C, plates were removed and kept at room temperature for an additional 24 h. This allows distinguishing QS cheats from cooperators based on differences in colony morphologies. While cooperators form raised colonies with fuzzy edges, *lasR* mutant colonies show characteristic colony flattening, sharper edges and surface iridescent sheen [25] (Fig. S1, available in the online version of this article). To change the viscosity and spatial structuring of the media, competition assays were performed in QSM supplemented with 0.5 % (semi-solid) agar [20].

### Antibiotic survival assays

To determine antibiotic survival of populations before or after competitions in QSM, 10 µl samples were spotted in triplicates onto LB agar plates supplemented with varying concentrations of antibiotics (Table 2). The inoculated plates were incubated at 30°C for 24 h and then at room temperature for an additional 24 h. The choice to run the antibiotic survival assays under conditions of 24 h at 30 °C, as opposed to the other experiments, was dictated by the need to ensure the reliability and accuracy of colony counting by agar spotting. At this lower temperature, cell growth is notably slower, which significantly mitigates the issues of overgrowth and the formation of fuzzy colony edges encountered at higher temperatures. These conditions facilitate a clearer distinction between individual colonies, thus enhancing the precision of our quantitative analyses. To obtain survival rate data, triplicate counts were averaged, and percentage of survival was calculated for each data point by the formula CFU(antibiotic)/CFU(no-antibiotic)×100.

**Table 2.** Minimum Inhibitory Concentrations *in vitro* (MICs)

Antibiotic	MIC for PA14 and Trojan	MIC for PA14^R^
Streptomycin	8 µg ml^−1^	2048 µg ml^−1^
Carbenicillin	53 µg ml^−1^	16384 µg ml^−1^
Ceftazidime	1 µg ml^−1^	1365 µg ml^−1^
Meropenem	0.5 µg ml^−1^	192 µg ml^−1^

### Murine chronic wound model assays

The murine chronic wound model utilized in this study has been previously described [26,27]. For the experiments herein, female Swiss Webster mice were anesthetized using intraperitoneal injection of ketamine and xylazine. The backs of the mice were shaved, and a full-thickness skin excision measuring 1.5 cm in diameter was administered. To cover the wounds, semipermeable polyurethane dressings were applied, followed by the inoculation of bacteria on top of the wounds, beneath the dressings. The initial inoculation of bacteria was performed at a concentration of 10^5^ CFU ml^−1^, with strains inoculated according to experimental group. For co-inoculated mixed infections, a Trojan to PA14^R^ ratio of 1 : 10 was used for initial inoculation. For delayed-mixed infections, PA14^R^ infection was allowed to establish for 72 h. At this point, the delayed-mixed infection groups received an additional 10^6^ CFU per 100 µl of Trojan strain, which corresponded to approximately 10 % of the wound burden of PA14^R^ at the 72 h mark post-infection. The remaining groups were sham inoculated with PBS. On day seven post-infection, the mice were euthanized. The wound beds were harvested and subjected to *ex vivo* treatment with either a vehicle control (PBS) or an antibiotic solution containing 1.75 mg ml^−1^ of streptomycin and 8 mg ml^−1^ of carbenicillin. The difference in dosage compared to *in vitro* experiments is due to the recalcitrant nature of biofilms in wounds, which reduces the efficacy of antibiotics even on genotypically susceptible infections [28]. Following antibiotic treatment, the wound beds were washed thoroughly to prevent antibiotic carryover, resuspended in PBS, and subsequently homogenized. Serial dilutions were prepared for CFU quantification, which was calculated per gram of tissue. To determine the population compositions, each homogenized tissue sample was plated on Pseudomonas Isolation Agar (PIA) or PIA supplemented with 200 µg ml^−1^ ceftazidime, which selects for the presence of the resistance plasmid.

### Statistics

To compare the means of two groups, we employed the t-test or the Mann-Whitney U test, depending on the homogeneity of variances between the groups. For the analysis involving multiple groups, linear models were initially fitted to the data; subsequent pairwise comparisons were conducted using the Tukey test. All statistical analyses were carried out, and figures were produced using R statistical software (http://www.R-project.org).

### Data availability

The data that support the findings of this study are available from the authors on reasonable request, see author contributions for specific data sets.

## Results

In this study, we investigated Trojan-mediated interventions against antimicrobial resistance, hypothesizing that the invasion of resistant populations (PA14^R^) by sensitive QS signal-blind cheats (Trojan cheats) could enhance antibiotic effectiveness. We first confirmed cheat invasion through competition assays in QS selective media, expecting these mutants to exploit cooperator-produced public goods without contributing themselves.

To examine the impact on antibiotic sensitivity of invaded populations we subjected them to various antibiotic doses. We predicted decreased resistance in invaded populations compared to uninvaded or purely resistant groups.

Considering the importance of environmental structure, we further explored Trojan strategy effectiveness in medium with added agar, simulating conditions of limited movement and increased viscosity typical of natural infections.

Lastly, we applied our approach to chronic wound infections, assessing invasion in an *in vivo,* and antibiotic effectiveness in an *ex vivo* chronic wound infection model.

### Growth and cheating in QSM

**Fig. 2.**
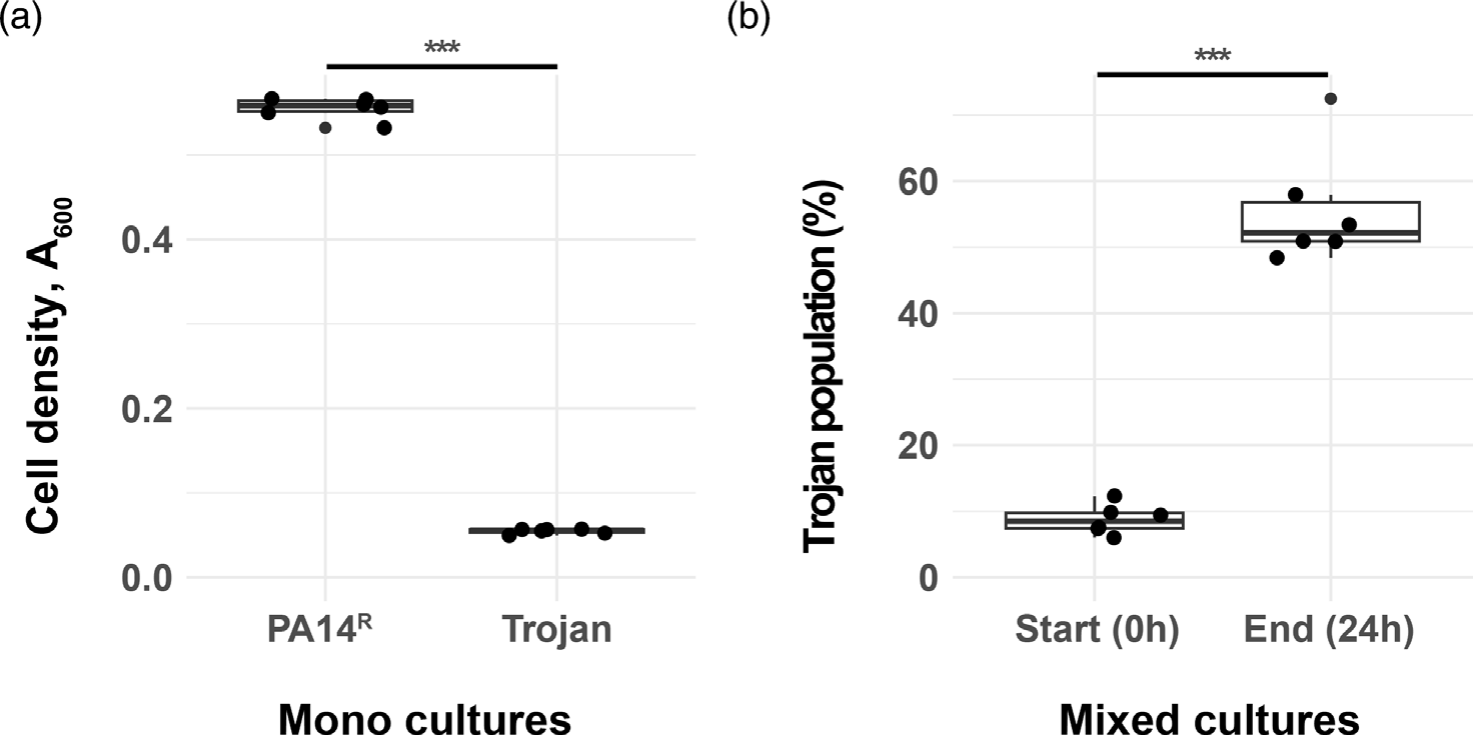
Growth and competitions in QSM. (a) Growth is measured as turbidity at 600 nm after 24 h incubation in QSM. Strains: PA14^R^: resistant PA14, Trojan: sensitive PA14ΔlasR cheat. *n*=6 populations. Two-sample t-test, t=93.188, d.f.=10, *P*<0.00001. (b) Percentage of Trojan population in mixed cultures at the start and at the end of fitness competitions. Two-sample t-test, t=−12.586, d.f.=10, *P*<0.00001. All mixed populations start with an initial ratio of 10 : 1 cooperator to cheat. *n*=6 populations.

We first confirmed that *lasR* encoded extracellular protease activity is required for optimal growth in QSM [11,24] by comparing growth of PA14^R^ and Trojan cheats in QSM without antibiotic selection. After 24 h incubation, Trojan monocultures were significantly lower in density than PA14^R^ monocultures (Fig. 2a). We obtained similar results when we cultivated antibiotic sensitive PA14^S^ and resistant Trojan^R^ strain variants in QSM (Fig. S2a).

In head-to-head competition assays, when Trojan cheats were co-inoculated with PA14^R^, they successfully invaded the populations, increasing in frequency, even when carrying the costly resistance plasmid (Figs 2b and S2b). We did not observe any significant impact of the resistance plasmid on monoculture growth (Fig. S2a). These results display that *lasR* regulated extracellular protease activity is required for maximal growth of PA14 in QSM. More importantly, these results show the capacity of Trojan cheats to exploit cooperation and invade mixed cultures. In addition, we showed that the presence of the antibiotic resistance plasmid is costly and can influence the level of cheat invasion.

### Antibiotic susceptibility of Trojan cheat invaded populations

**Fig. 3.**
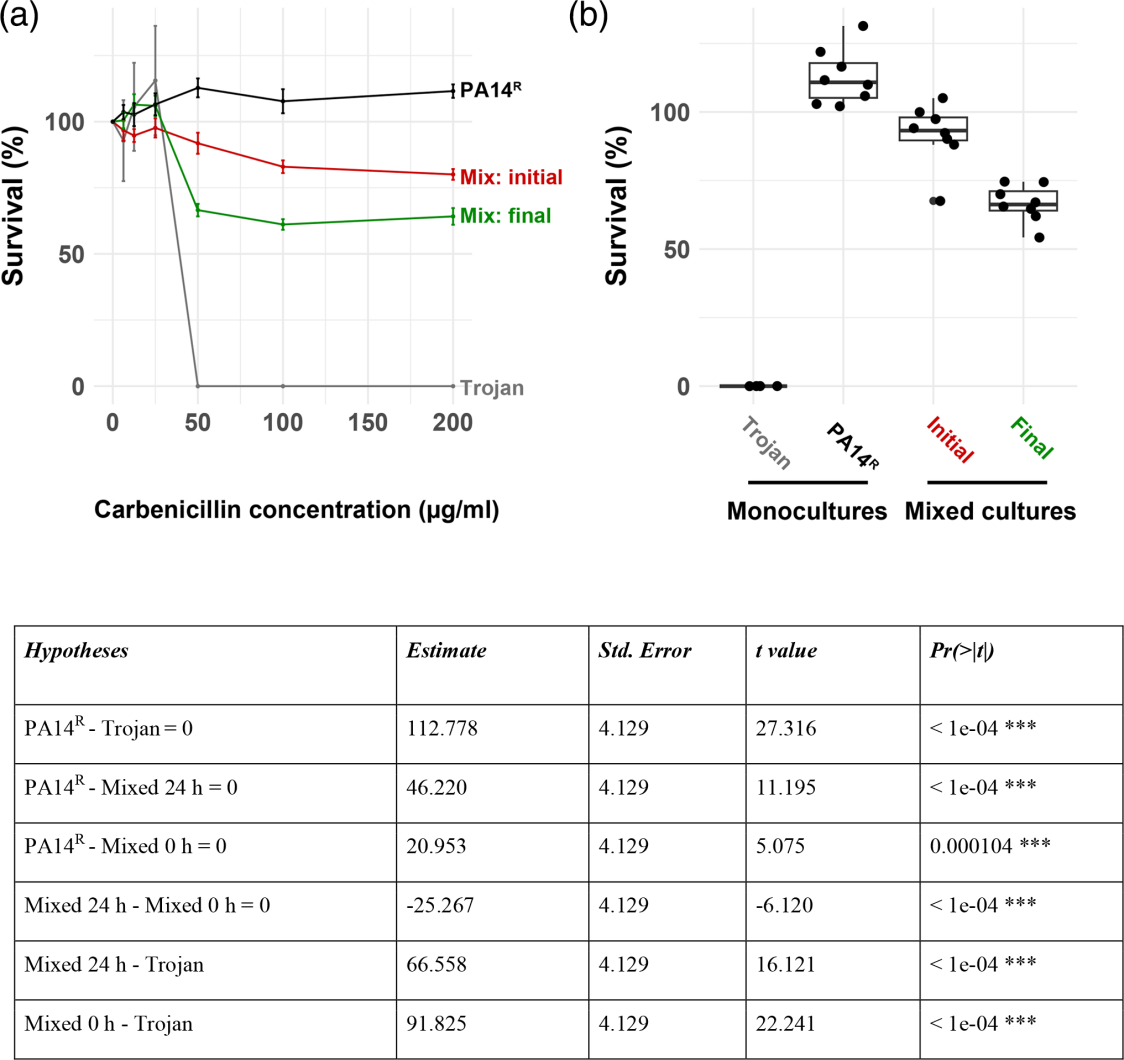
Antibiotic susceptibility of Trojan cheat invaded populations.Cultures grown in QSM without antibiotic were spotted on antibiotic agar media and survival percentages were determined from CFUs. (**a**) Antibiotic survival curves: black: PA14^R^ monocultures at 24 h; grey: Trojan cheats at 24 h; red: mixed populations at the beginning of competition; green: mixed populations after 24 h competition in QSM. All mixed populations started with an initial ratio of 10 : 1 cooperator to cheat. *n*=8 populations per condition. Error bars indicate standard error of the mean. (**b**) Antibiotic survival before and after Trojan invasion. Single and mixed populations exposed to 50 µg ml^−1^ carbenicillin. Mixed populations were assayed at the start (0 h) and at the end (24 h) of competitions, whereas monocultures were assayed after 24 h growth in QSM. *n*=8 populations. Error bars indicate standard error of the mean. The table shows the results of post-hoc pairwise comparison of survival at 50 µg ml^−1^ carbenicillin. MIC_Trojan_=50 µg ml^−1^, MIC_PA14R =_ 16384 µg ml^−1^ .

To test the efficacy of antibiotics against Trojan invaded and uninvaded populations, we compared population survival at the beginning and at the end of competitions after exposing to varying doses of carbenicillin. We hypothesized that following the expansion of the sensitive Trojan population, mixed populations would show increased susceptibility to antibiotics compared to uninvaded PA14^R^ populations. As shown in Fig. 3, our results confirmed this hypothesis; mixed cultures of PA14^R^ and Trojan were more susceptible than pure PA14^R^ monocultures. Notably, while PA14^R^ monocultures were able to resist carbenicillin doses up to 200 µg ml^−1^, sensitive Trojan monocultures could not grow above doses higher than their MIC_Trojan_ (50 µg ml^−1^) (Fig. 3a). Consequently, this led to a significant decline in survival rates for susceptible and mixed populations when exposed to carbenicillin at the MIC_Trojan_ (Fig. 3b). Moreover, post-invasion mixed cultures were even more susceptible compared to their initial state (Fig. 3b). A similar reduction in survival rates was observed when we replicated the experiments using different antibiotics to which the pAMBL2 plasmid confers resistance (Fig. S3). These findings emphasize the potential to induce partial antibiotic sensitivity in target populations as a result of successful Trojan cheat invasions. As a result, Trojan cheat invasion has the potential to enhance the efficacy of antibiotics.

### Increased viscosity hinders Trojan cheat effectiveness

**Fig. 4.**
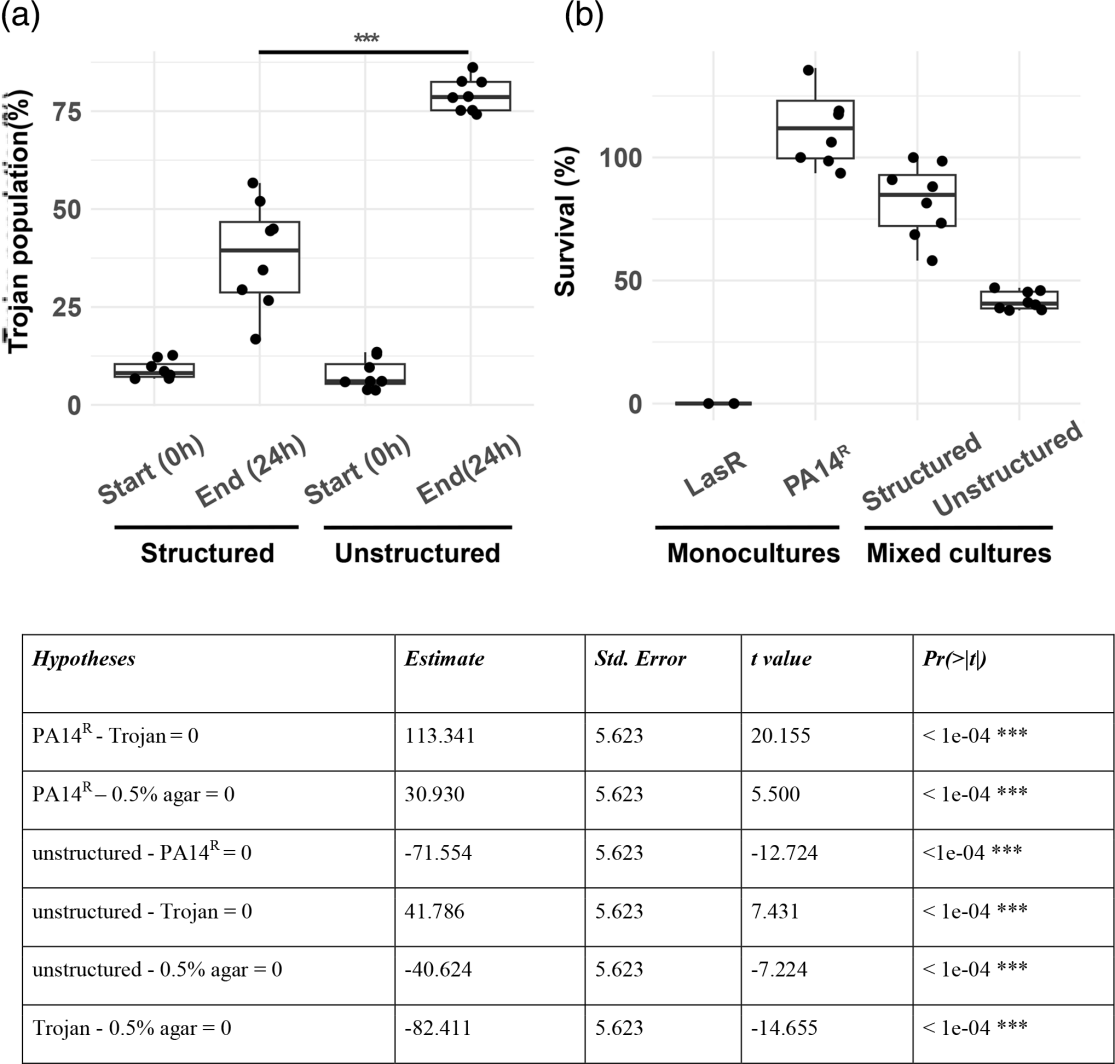
Spatially structured media hinders cheat invasion and antibiotic effectiveness. (a) Invasion of Trojan cheats in spatially structured and unstructured conditions. Competition assays were performed in the presence and absence of agar in QSM without antibiotic selection for 48 h. *n*=8 populations per condition. Two-sample t-test, t=−8.1156, d.f.=14, *P*<0.00001. (**b**) Antibiotic survival of Trojan cheat invaded populations exposed to 12.5 µg ml^−1^ streptomycin. The table shows the results of post-hoc pairwise comparison of survival rates at 12.5 µg ml^−1^ streptomycin. MIC_Trojan_=8–12 ug ml^−1^, MIC_PA14R =_ 2048 µg ml^−1^. All mixed populations start with an initial ratio of 10 : 1 cooperator to cheat.

In natural infections, where populations are likely to be more structured than in our liquid media conditions, accessibility to public goods may be limited for invading Trojan cells [20]. To assess the effectiveness of Trojan invasion in spatially structured populations, we conducted competitions in QSM supplemented with 0.5 % (semi-solid) agar. This medium composition is known to influence cell mobility and the diffusion of public goods [20]. In comparison to unstructured media, Trojan cheats invaded the populations to a lesser degree in viscous media (Fig. 4a). This reduced invasion observed in viscous media also translated into a decreased efficacy of antibiotic treatment. Mixed populations in viscous media were less susceptible to streptomycin treatment than mixed populations in unstructured media (Fig. 4b). It’s important to note that the control group (unstructured), which was subject to the same conditions as seen in a prior experiment (Fig. 2b), displayed a significant variance in cheat invasion levels (Fig. 4a). This discrepancy not only underlines the variability inherent in competition experiments but also significantly influences the survival rates post-antibiotic treatment (Fig. 4b).

### Trojan strain can invade infections *in vivo*

**Fig. 5.**
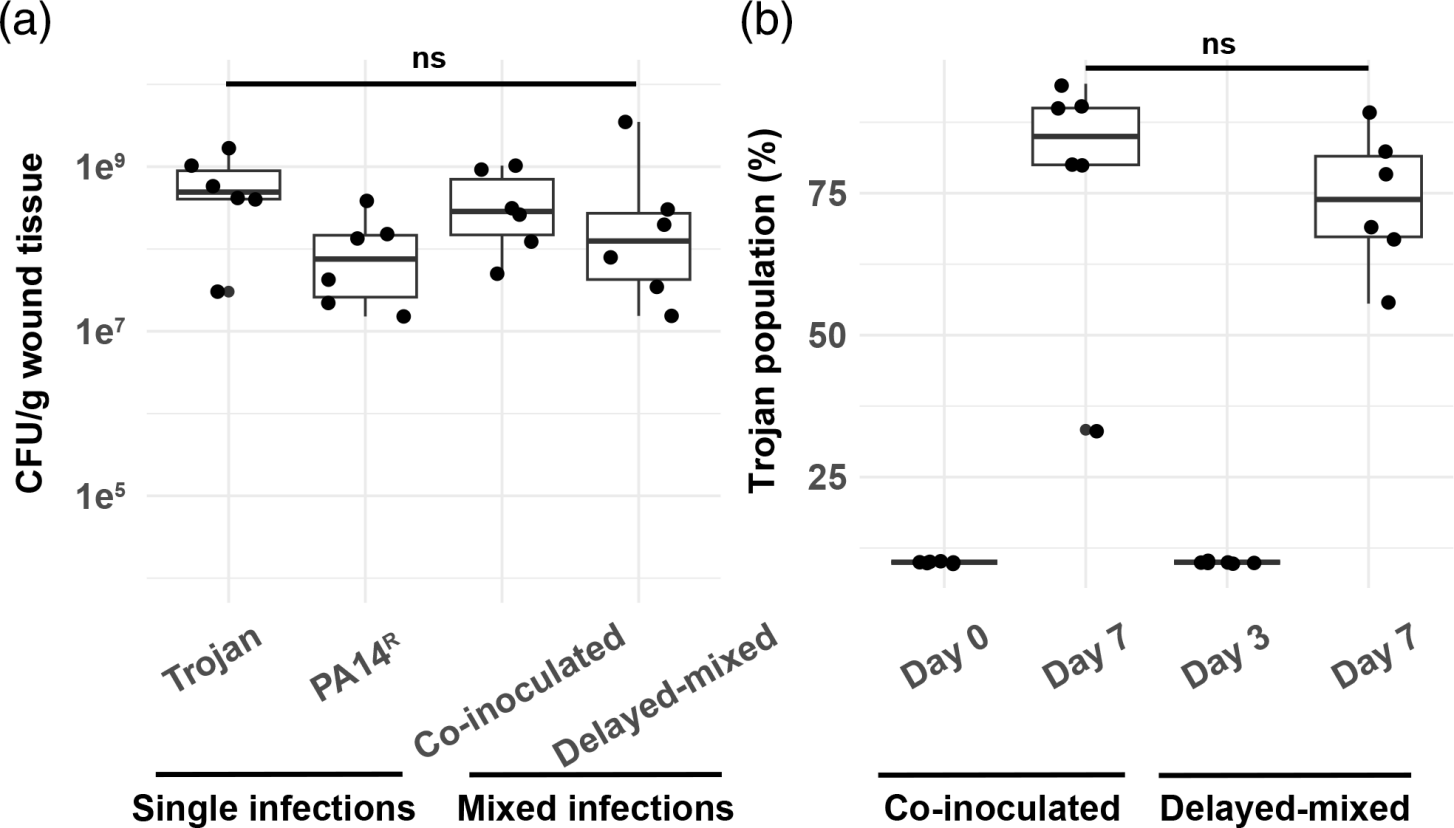
Trojan cheats can invade resistant infections *in vivo.* (a) Total bacterial load of the wound extracts at 7 days as CFU per gram of wound tissue. *n*=6 per group. Kruskal-Wallis chi-squared=5.8933, df=3, *P*=0.1169. (**b**) Trojan population percentage was determined after collection of wounds on day seven from CFU per gram tissue measurements. All mixed populations start with an initial ratio of 10 : 1 PA14^R^ to Trojan cheat at the time of mixing. *n*=6 populations. Trojan invasion levels are not significantly different between different introduction times. Mann-Whitney U test W=10, *P*=0.2281.

After confirming Trojan invasion in unstructured and viscous *in vitro* environments, we proceeded to examine the population dynamics of the Trojan strain in combination with PA14^R^ in an *in vivo* chronic wound infection assay. Following 7 days of infection, we observed no significant difference in the total bacterial load in the wounds between single and mixed infections (Fig. 5a), nor did we observe abnormal pathology as a result of the increased bacterial load from the Trojan cheat alone. The attenuation of virulence in *in vivo* infections with quorum sensing mutants such as *lasR* has been shown previously [16]. However, in both co-inoculated populations and delayed-mixed populations, the Trojan strain successfully invaded up to 75 % of the populations by the end of the assay (Fig. 5b). These results demonstrate the invasive capability of QS mutants in mixed infections. Importantly, our findings also highlight the invasive ability of QS mutants even when introduced to an already established PA14^R^ infection.

### Antibiotic treatment reduces bacterial load in Trojan-invaded populations *ex vivo*

Having demonstrated that the Trojan strain could invade PA14^R^ infections, we proceeded to investigate the antibiotic susceptibility of these invaded populations. Antibiotic susceptibility was determined using *ex vivo* incubation of excised wound tissue to limit experimental variation caused by the murine host, i.e. leakage of topical antibiotic from beneath the wound dressing, distribution of antibiotic from topical treatment into systemic circulation, and degradation of antibiotics by host components. *Ex vivo* conditions provided control and reproducibility for these experiments. To this end, fewer animals can be used, and by dividing the excised wound tissue, each animal acts as its own control to compare bacterial load following vehicle vs. antibiotic treatment.

**Fig. 6.**
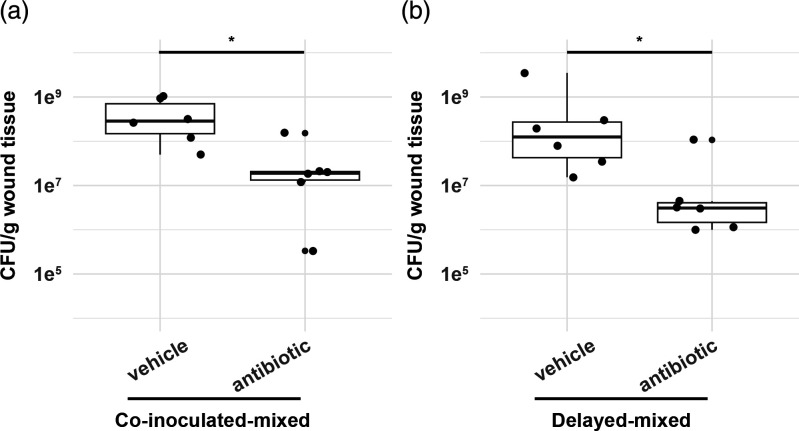
Trojan-invaded mixed infections are more susceptible to antibiotic treatment. Infected wound beds were harvested and treated *ex vivo* with antibiotics (1.75 mg ml^−1^ streptomycin +8 mg ml^−1^ carbenicillin) or with PBS. Total bacterial load was expressed as CFU per gram wound tissue. (**a**) Total bacterial load of antibiotic-treated wound extracts obtained from infections co-inoculated at time 0. Mann Whitney-U test W=2, *P*=0.01307 (b) Total bacterial load of antibiotic-treated wound extracts obtained from delayed-mixed infections. *n*=6 populations. Mann Whitney-U test W=3, *P*=0.02024.

Similar to *in vitro* observations, we expected that the increased frequency of the invading Trojan subpopulations would lead to a reduction in the total bacterial load in mixed infections following antibiotic treatment. In single infections of the Trojan strain, we observed higher susceptibility to antibiotics, resulting in a three-log reduction in bacterial load (Fig. S4b). In contrast, PA14^R^ infections displayed resistance to antibiotic treatment (Fig. S4a). However, unlike our *in vitro* assays, complete killing of the sensitive populations proved challenging due to the resilient nature of wound extracts. When we treated the wound beds collected from both initially co-inoculated and delayed-mixed infections, we observed a 1–2 log decrease in total bacterial load compared to uninvaded single PA14^R^ infections (Fig. 6). The reduction in total bacterial load can be attributed to the elimination of the susceptible Trojan subpopulation, as we noted a comparable decrease in Trojan load in mixed populations (Fig. S5b).

These results demonstrate that Trojan invasion *in vivo* can induce partial antibiotic sensitivity in an otherwise resistant infection, highlighting the potential for manipulating antibiotic resistance of a target population through Trojan invasion.

## Discussion

We provide experimental evidence supporting the Trojan horse approach as a potential medical intervention strategy. Following the successful invasion of Trojan strains when introduced into both *in vitro* and *in vivo* populations of PA14^R^ (Figs2, 4  5), a larger bacterial population was eliminated with antibiotics in comparison to clonal populations and single infections of PA14^R^ (Figs3  6). While we observed successful invasion by cheats under the tested conditions, it is crucial to take into account various factors known to influence cooperator-cheat dynamics [4,10, 11, 13, 14, 20, 29] when considering the viability of this strategy. These factors include population viscosity, the relative frequency of cheats, population density, the nature of selection, and the mechanism of cheating.

Our findings from the *in vivo* experiments (Fig. 5) not only confirmed earlier observations of *lasR* cheat invasions of PA14 [16], but also offer new insights into potential applications of Trojan cheats to combat infections. We demonstrated the feasibility of introducing partial antibiotic sensitivity to a target population of chronic PA14 infection, leading to increased antibiotic susceptibility in that population (Fig. 6). Moreover, we showed for the first time, to our knowledge, the possibility of introducing Trojan cheats into an established infection. In a therapeutic intervention, a Trojan cheat would be introduced in this fashion to an ongoing infection, rather than co-occurring from the onset, thus emphasizing the application potential of this therapeutic strategy.

While we successfully demonstrated the potential of employing Trojan cheats, it’s important that the bacterial load after antibiotic treatment remained substantial both *in vitro* (Fig. 3) and *in vivo* (Fig. 6). The *in vitro* experiments showed notable variability in the levels of cheat invasion under identical conditions, influencing the survival rates post-antibiotic application (see variations between Figs2  4, unstructured group). Similarly, *in vivo* experiments revealed that, despite significantly reducing the genotypically susceptible Trojan population, the remaining bacterial load still surpassed the clinically accepted infection threshold for wounds, which is generally established at 10^5^ CFU g^−1^ of tissue [29,30] (Figs 6, S4 and S5). Therefore, certain limitations must be addressed before advancing to clinical applications. These necessary improvements include improving invasion levels, and improved targeting of the resident population.

One approach to enhance cheat invasion is through engineering more effective invaders. While single-trait mutants serve as a promising starting point, theoretically, it is possible to create even better invaders that exploit multiple cooperative traits simultaneously [31]. Using such enhanced cheats would have the added benefit of further reduced virulence, as many cooperative traits are associated with virulence [32]. To improve the Trojan strain that we used in this work, one could consider using double QS mutants, such as *lasR/rhlrR,* instead of the single *lasR* mutant. Furthermore, mutants capable of exploiting both QS and other social traits, like siderophore production [12,15], could be explored. Depending on the specific mechanisms of cooperation and cheating, increasing the extent of cheating could also be considered as a viable option [33]. Additionally, genome reduction strategies could be applied to enhance overall strain fitness and invasive capabilities [34]. Since biofilms have been extensively documented to increase antibiotic tolerance [35,36], one can also adopt several approaches to disperse biofilms to improve cheat invasion and antibiotic effectiveness. These approaches include mechanical removal or using molecular dispersal agents like matrix-degrading enzymes, anti-biofilm peptides, and dispersal molecules to disrupt biofilms [37].

One major concern in almost all biocontrol methods is the potential restoration of virulence once the target population is invaded by external agents. In the case of antibiotic sensitivity introduction, the primary worry is the Trojan population acquiring resistance through horizontal gene transfer. Preventing this scenario involves implementing fail-safe mechanisms while constructing Trojan cheats. These mechanisms could include synthetic inducible systems for toxin-antitoxin or phage lytic protein expression to trigger programmed self-destruction upon invasion and antibiotic treatment [38,39].

In conclusion, this study presents initial experimental evidence demonstrating the potential application of cheats to socially engineer and deliver medically advantageous alleles, such as antibiotic sensitivity, to target populations. The aforementioned technical improvements and the development of robust and secure delivery strategies will facilitate the clinical implementation of Trojan strains as a novel alternative strategy to fight infections.

## supplementary material

10.1099/mic.0.001454 Uncited Fig. S1.

10.1099/mic.0.001454 Uncited Table S1.

## Abbreviations

A600: optical density 600 nm
CFU: colony-forming unit
Gm: gentamicin
KB: King’s broth media
MIC: minimum inhibitory concentration
PBS: phosphate buffered saline
QS: quorum sensing
QSM: QS medium
WT: wild-type
