# Supplementary material for: Exploiting cooperative pathogen behaviour for enhanced antibiotic potency: A Trojan horse approach
Source: Microbiology (Reading). 2024 Apr 30;170(4):001454. doi: 10.1099/mic.0.001454 (PMC11084615; doi:10.1099/mic.0.001454)
Supplement: Uncited Fig. S1. [file mic-170-01454-s001.pdf]

SUPPLEMENTARY INFORMATION

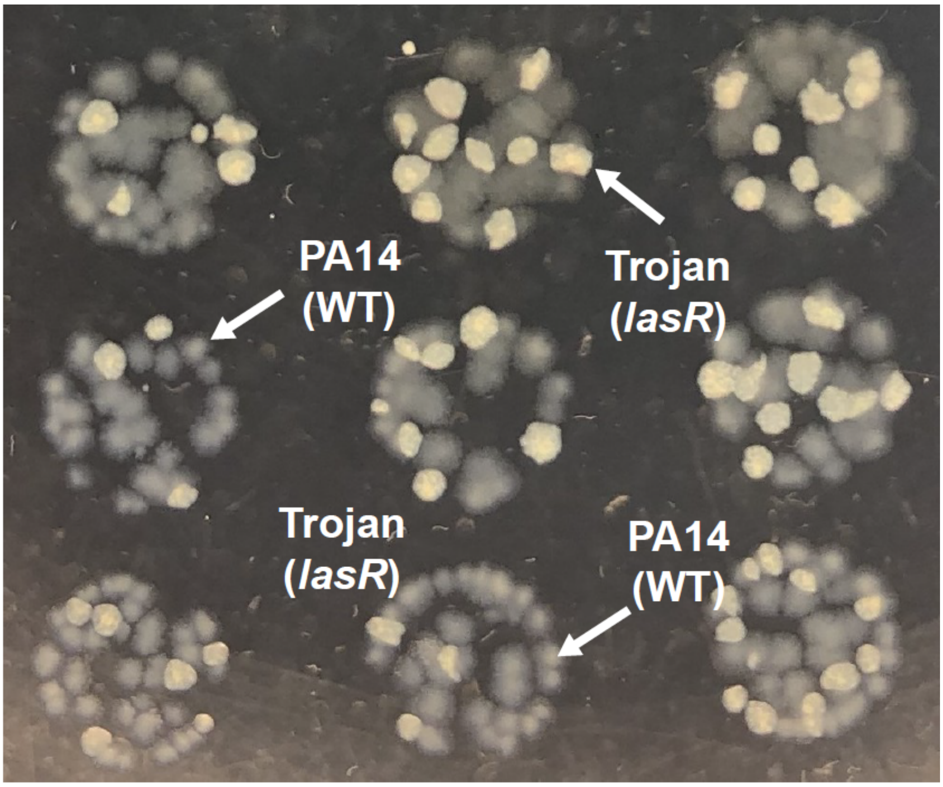

Figure S1. Distinguishing Trojan and PA14 strains on LB Agar plates. Following a 24 hour incubation at 37°C and an additional 24 hours at room temperature, the distinct colony morphologies of QS cheats and cooperators become apparent on LB agar plates.

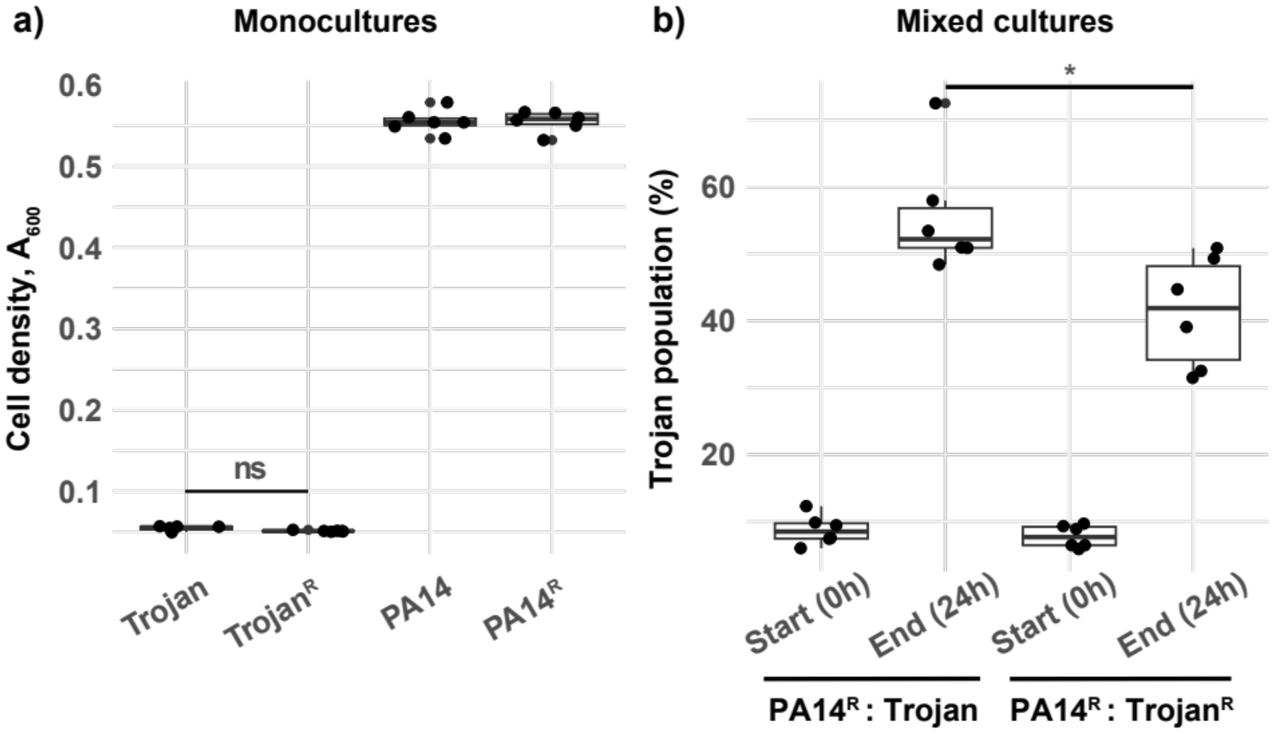

Figure S2. Growth and invasion in plasmid bearing strains. *a) Monoculture growth was measured at 600 nm after incubating in QSM for 24 hours. Strains: Trojan: antibiotic sensitive Trojan cheat, Trojan^R: antibiotic resistant Trojan cheat, PA14: antibiotic sensitive PA14, PA14^R: antibiotic resistant PA14. n=6 populations. PA14^R vs PA14 (t-test, t = -0.021053, df = 10, p = 0.9836); Trojan^R vs Trojan (Mann-Whitney U test, W = 29, p = 0.0927) b) Percentage of Trojan population in mixed cultures at the start and at the end of fitness competitions. Two-sample t-test, t = -2.8972, d.f. = 10, p = 0.01591. All mixed populations start with an initial ratio of 10:1 cooperator to cheat. n = 6 populations.*

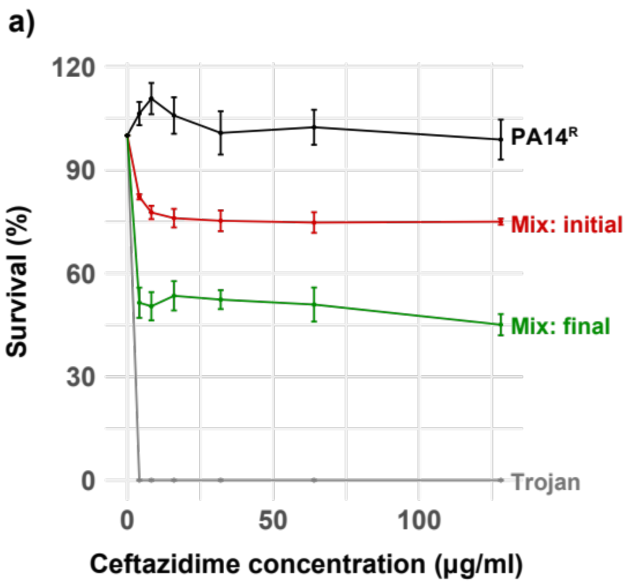

Figure S3. Survival curves against ceftazidime and meropenem. *Bacterial cultures grown in QSM without antibiotics were placed on antibiotic agar media, and the percentage of survival was determined by counting the number of CFUs. Antibiotic survival curves: Black: $PA14^R$ monocultures at 24 h; gray: Trojan cheats at 24 h; red: Mixed populations at the start of competition; green: Mixed populations after 24 h competition in QSM. All mixed populations start with an initial ratio of 10:1 cooperator to cheat. n = 8 populations per condition. Error bars indicate standard error of the mean. Ceftazidime MIC_{Trojan} = 1 µg/ml, MIC_{PA14R} = 1365 µg/ml. Meropenem MIC_{Trojan} = 0.5 µg/ml, MIC_{PA14R} = 192 µg/ml.*

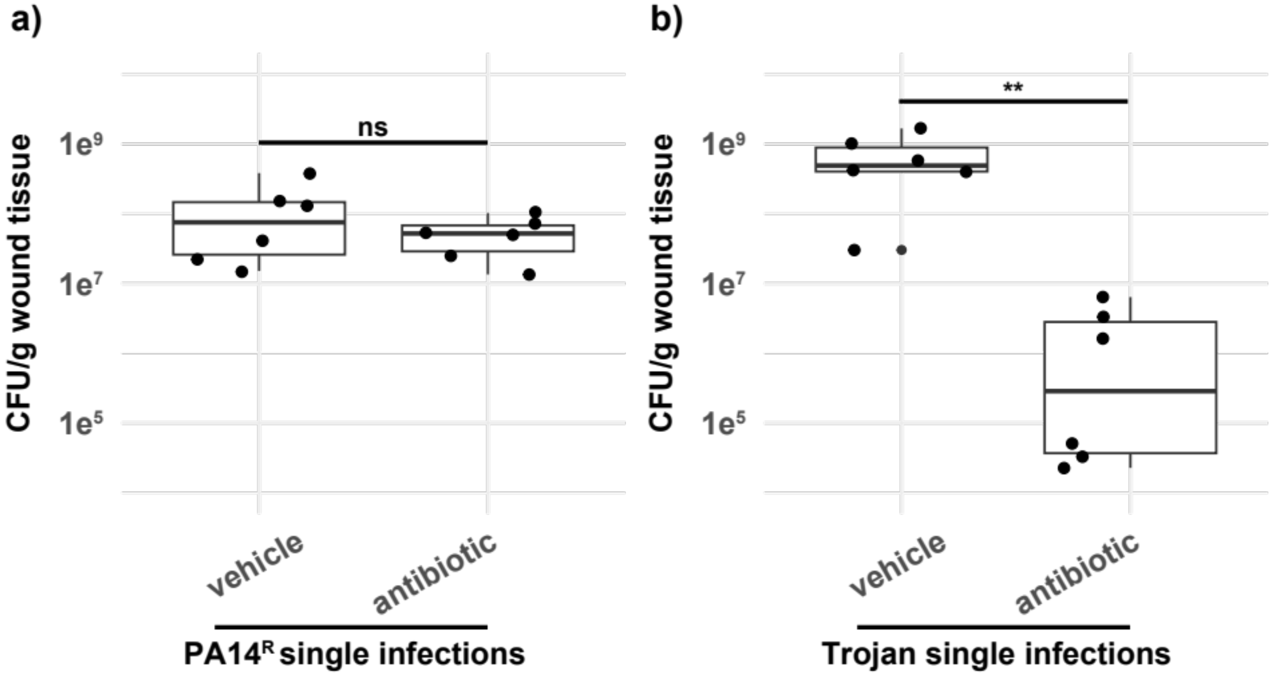

Figure S4. Trojan cheats are more susceptible to antibiotic treatment than PA14^R. *Infected wound beds were harvested and treated ex vivo with antibiotics (1.75 mg/mL streptomycin + 8 mg/mL carbenicillin) or with PBS. Total bacterial load was expressed as CFU/g wound tissue. n= 6 populations for all experiments. a) Bacterial load after antibiotic treatment of PA14^R infected wound extracts ($W = 14$, $p = 0.5752$). b) Bacterial load after antibiotic treatment of Trojan infected wound extracts ($W = 0$, $p = 0.005075$).*

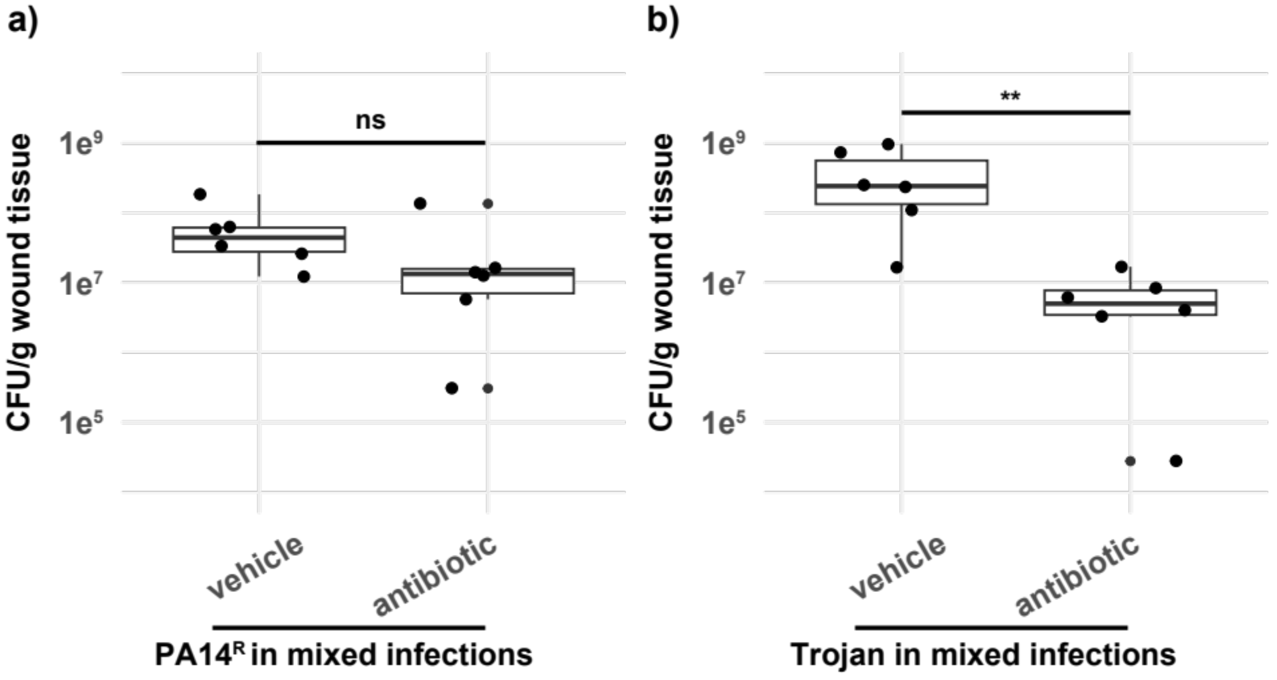

Figure S5. Only Trojan subpopulation is significantly reduced by antibiotic treatment in mixed infections. *Infected wound beds were harvested and treated ex vivo with antibiotics (1.75 mg/mL streptomycin + 8 mg/mL carbenicillin) or with PBS. Bacterial load was expressed as CFU/g wound tissue. n = 6 populations. a) PA14^R load in mixed infections after antibiotic treatment (W = 8, p = 0.1282). b) Trojan load in mixed infections after antibiotic treatment (W = 1, p = 0.008239).*
